# On the limits of marginal protein folding stability: methodological goals and drawbacks

**DOI:** 10.1007/s00894-026-07034-6

**Published:** 2026-10-27

**Authors:** David Ferreiro, Valeria Zoni, Miguel Arenas

**Affiliations:** 1 CINBIO, Universidade de Vigo, 36310 Vigo, Spain; 2 Department of Biochemistry, Genetics and Immunology, Universidade de Vigo, 36310 Vigo, Spain

**Keywords:** Protein stability, Protein evolution, Protein modeling, Marginal stability, Structural constraints

## Abstract

**Context:**

Why do proteins remain at the edge of stability rather than evolving toward maximal thermodynamic robustness? Can we trust the computational methods that inform about when and how stability falls off that edge? Marginal stability, the confinement of protein folding free energies to a window of approximately 5–15 kcal/mol, is broadly observed in biophysics, and its mechanistic causes, involving evolutionary and biophysical properties, remain under debate and require accurate predictions. In this regard, protein folding stability is currently predicted using both traditional structure-based homology-modeling methods and modern sequence-based deep-learning methods. However, comparisons among these methodologies remain scarce.

**Methods:**

Using four viral proteins of biomedical interest and considering that rapidly evolving viral proteins provide a useful system for understanding thermodynamic tolerance under the competing demands of structural integrity and adaptive flexibility, we compared predicted protein stability from both approaches across independent random mutational trajectories and naturally occurring sequence variants available in public databases. The results showed a general methodological impasse. As random mutations accumulated, structure-based methods predicted a progressive decline in stability toward collapse, a pattern driven by the enforced conservation of protein structure. In contrast, sequence-based methods failed as sequences moved into uncharted regions of parameter space. Together, protein stability landscapes predicted using current computational approaches are inherently shaped by technical limitations, leading to uncertainty in the evaluation of marginal stability and stability changes associated with biological processes.

**Supplementary Information:**

The online version contains supplementary material available at https://doi.org/10.1007/s00894-026-07034-6.

## Introduction

Structural proteins generally adopt well-defined structures that remain stable over long evolutionary timescales, during which protein function is preserved through selective constraints [1–5]. A standard quantitative measure of this stability is the folding free energy, which is usually quantitatively evaluated by the Gibbs free energy difference (Δ*G*) between folded (native) and unfolded states [2, 6]. Thus, to maintain a population of functional proteins, the native state of a protein can evolve under selection to reach a new state often with lower free energy than unfolded or misfolded states [7, 8]. In the context of molecular evolution, thermodynamic stability is usually considered one of the primary determinants of fitness [3, 9], as stable folding is a prerequisite for structural proteins to preserve their specific functions [10, 11]. For example, multiple human diseases result from failure of protein folding, including cystic fibrosis, Alzheimer disease, and Parkinson disease [8]. Also, some disease-associated mutations affect protein stability rather than directly disrupting function, thereby decreasing the cellular abundance of the folded functional form [12–14]. However, the relationship between stability and fitness is not linear. Although higher stability is often assumed to correlate with increased fitness, natural selection does not drive proteins toward maximal stability. Protein active sites and binding interfaces frequently contain structural features, such as uncompensated charges or solvent-exposed hydrophobic patches, that are required for protein activity but inherently destabilize the protein structure [15, 16]. Substitutions that remove these destabilizing features may increase stability, sometimes at the cost of functional loss. Comparative analyses of diverse protein families have shown that, rather than being highly stable, proteins are generally marginally stable, with folding free energies under physiological conditions usually falling within a relatively narrow range of approximately 5–15 kcal/mol, regardless of protein size or function [17, 18]. This marginal stability may not represent a limitation but rather a requirement for many dynamic molecular activities. For example, conformational flexibility is essential for proteins involved in regulated unfolding (e.g., during membrane transport) as well as for functional conformational changes such as allosteric regulation [19]. Excessive stability can lock proteins into rigid conformations, thereby hindering these essential conformational transitions [20, 21]. Thus, marginal stability may reflect a trade-off between the accumulation of slightly destabilizing or stabilizing mutations through genetic drift and the selective pressure to maintain a required fraction of functional folded proteins.

Exploring the limits of protein folding stability remains a major methodological challenge. Experimental approaches such as deep mutational scanning (DMS) provide high-resolution maps of fitness landscapes, yet their high computational cost and time requirements limit their scalability. Consequently, the field has increasingly relied on computational mutational scanning (CMS) to simulate evolutionary trajectories and evaluate the impact of random mutational drift versus selective filtering. However, these computational methods, ranging from empirical force fields to deep-learning protein language models, often yield contrasting predictions and can introduce biases that distort our understanding of marginal protein stability.

In viral protein evolution, understanding and evaluating marginal stability is particularly important because many viral proteins rapidly accumulate mutations while maintaining stability and activity and, importantly, can develop immune escape and resistance to antiviral treatments [11, 22, 23]. Therefore, predicting a priori whether a viral protein exhibits marginal stability could help to obtain stability-based fitness landscapes and identify mutations that preserve protein fitness while potentially conferring resistance to antiviral therapies. Motivated by these applications and considering that traditional structure-based homology-modeling (HM) methods and modern sequence-based machine-learning (ML) methods used for predicting stability in CMS show important goals and drawbacks, we evaluated both methods for the prediction of folding stability in rapidly evolving proteins. Moreover, we systematically assessed the inherent methodological limitations of both structure-based HM and sequence-based ML methods, showing how their intrinsic biases can propagate into protein stability predictions along evolutionary trajectories in rapidly evolving viral systems.

## Materials and methods

We explored the effects of mutations and drift on protein folding stability through exhaustive CMS applied to four relevant viral proteins, including the human immunodeficiency virus (HIV) protease (HIVpr), the HIV matrix (HIVma), the hepatitis C virus (HCV) NS3 protein (HCVns3), and the influenza virus NS1 protein (FluNS1). Starting from a protein structure (*T*_*0*_) representative of the protein family (Table S1, Supplementary Material), we simulated 50 independent mutational trajectories where each residue of the sequence was replaced by a random amino acid and iterating it until every position was mutated at least once. To compare the folding stability predicted along the evolutionary trajectories with that predicted from real data, for each protein, we also retrieved 100 real sequences with high diversity from public databases that include Los Alamos [24, 25], Stanford HIV drug resistance database (HIVdb) [26], and NCBI [27]. For the HIVpr, we additionally retrieved 50 real protein sequences of HIV-2 protease (HIV-2pr), which share a ~ 50% identity with the HIV-1 protease sequence of the corresponding structure collected from the Protein Data Bank (PDB). The accession codes of these sequences are provided in Table S1, Supplementary Material.

Next, we predicted the folding stability of the simulated and real protein sequences using different approaches. The structure-based HM approach implemented in the well-established framework *FoldX* [28, 29] and the sequence-based ML approach implemented in the also well-established framework *THPLM* that includes a transformer-based hidden protein language model [30]. The “Discussion” section includes a rationale for the selection of these representative frameworks. For the HM approach, we predicted the structures of mutated protein variants from the evolutionary trajectories and real datasets by homology modeling using *Modeller* [31, 32], with the corresponding selected PDB structure (*T*_*0*_) used as the template (Table S1). The prediction of folding stability with *FoldX* relies on a rigid-solid approach where the protein backbone is kept relatively fixed, while the amino acid side-chains are allowed to rotate and find the most energetically favorable conformations upon mutation [28, 29]. Regarding the ML approach, only the sequence of mutated variants from the evolutionary trajectories and real datasets are required to make the predictions. Of note, sequence-based ML *THPLM* leverages pretrained protein language models (PLMs) [33], to extract high-dimensional representations of amino acid sequences and the encoder converts them into embeddings. By averaging these embeddings across the sequence to reduce position-specific bias and calculating the difference between the mutant and wild-type vectors, the framework feeds a condensed representation into a convolutional neural network decoder to predict folding stability [30].

We compared the folding stability of simulated and real protein sequences predicted with both methods at an equivalent evolutionary scale of amino acid replacements with respect to *T*_*0*_. We also investigated the effect of the structural template by comparing the stability predictions for the HIVpr real datasets using the original template, *T*_*0*_ (1KJH [34]), and two alternative templates (*T′*_*0*_). Specifically, we considered a structure differing from *T*_*0*_ by 14 amino acid substitutions (*T′*_*0*_, HIV-1 3IXO [35]) (Table S1) and a more divergent structure differing by 52 amino acid substitutions (*T′*_*0*_, HIV-2 3S45 [36]) (Table S1). For each of these alternative structures, we predicted stability again using the two approaches described above.

Additionally, we evaluated the structural effects of mutations using the root mean square deviation (RMSD) and the predicted template modeling (pTM) score for *FoldX* and *THPLM*, respectively. These metrics provide complementary information on the structural effects of mutations. In particular, RMSD quantifies the deviation in atomic positions between protein structures predicted by homology modeling from mutated sequences and the reference template *T*_*0*_. Likewise, the pTM score provides a measure of confidence in the overall topology of the predicted protein structure. In this regard, we used *ESMFold* [33] to predict the structures of simulated and real protein sequences together with their corresponding pTM scores. We predicted structures for 10 independent simulated evolutionary trajectories as well as for all real sequence datasets, resulting in more than 6500 predicted structures. Specifically, the analyses included 150 real sequences and 990 simulated mutants for HIVpr, 100 real sequences and 1150 simulated mutants for HIVma, 100 real sequences and 1910 simulated mutants for HCVns3, and 100 real sequences and 2020 simulated mutants for Flu NS1.

## Results

**Fig. 1:**
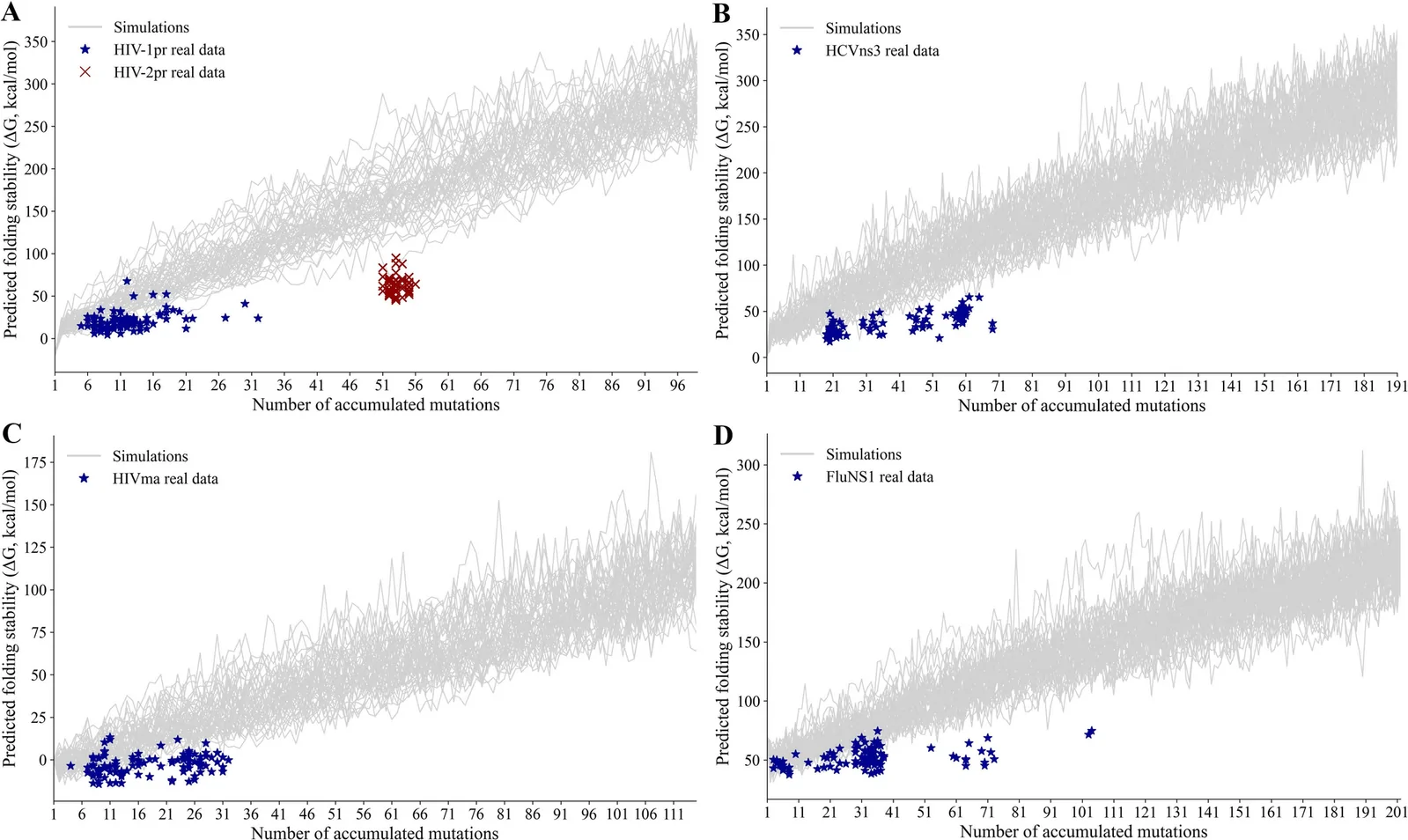
Protein folding stability of simulated mutational trajectories and real sequence data predicted using a structure-based HM approach. Relationship between the number of accumulated mutations and the protein folding stability (Δ*G*) predicted with *FoldX*. Panels show results for **A** HIVpr, **B** HCVns3, **C** HIVma, and **D** FluNS1 proteins. Simulated evolutionary trajectories are shown in gray, whereas real sequence data are represented by blue stars. In panel **A**, blue stars correspond to HIV-1pr sequences and red crosses to HIV-2pr sequences

**Fig. 2:**
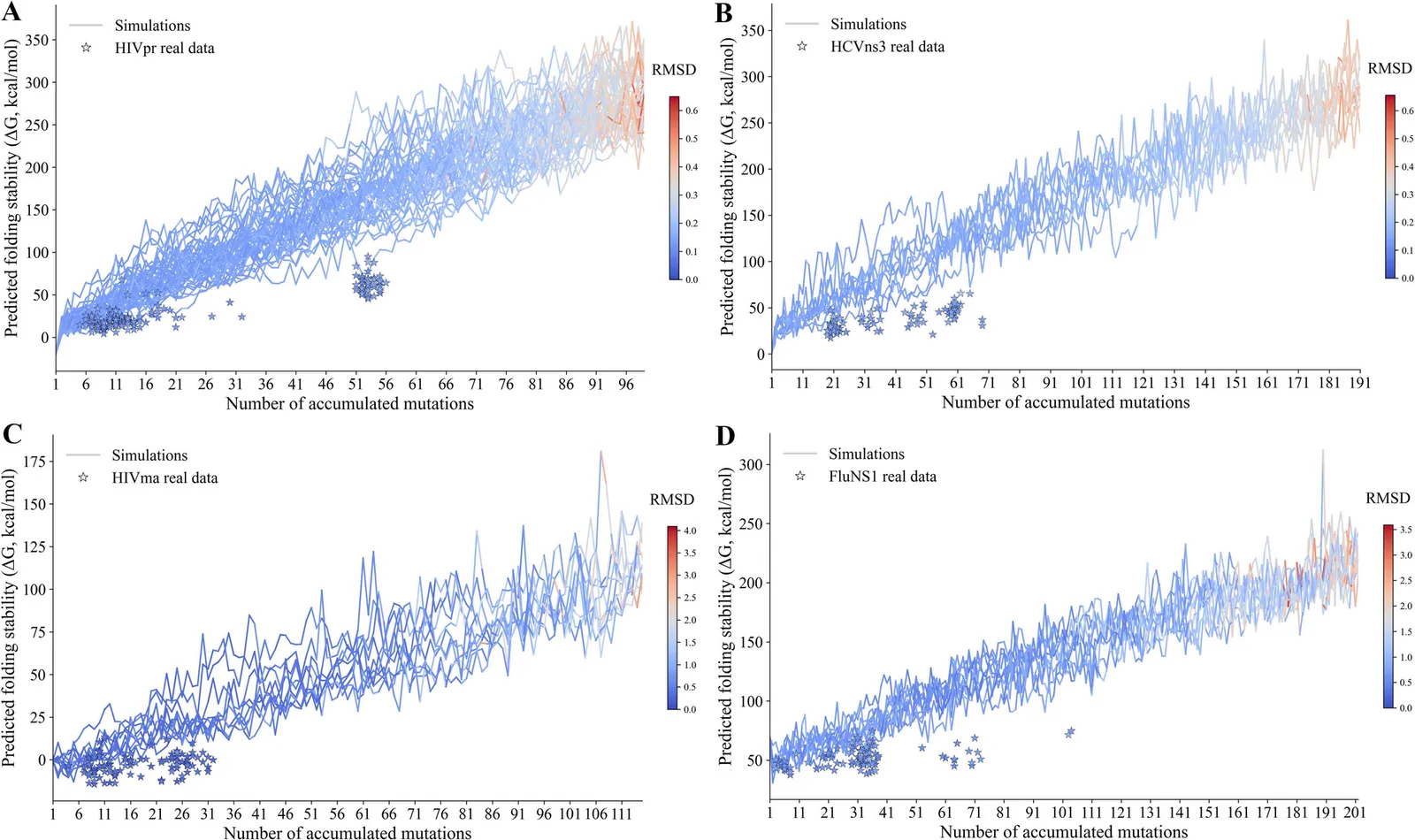
Structural divergence of simulated mutational trajectories and real sequence data relative to the reference structure. Relationship between the number of accumulated mutations, protein folding stability (Δ*G* predicted with *FoldX*), and RMSD relative to the reference structure (*T*_*0*_) for simulated and real data. Panels show predictions for **A** HIVpr, **B** HCVns3, **C** HIVma, and **D** FluNS1 proteins. Simulated evolutionary trajectories are colored according to their RMSD from the reference structure *T*_*0*_, with red indicating higher structural divergence than the reference. Real sequence data (stars) are displayed using the same RMSD color scale relative to *T*_*0*_

Using the structure-based HM approach (with *FoldX*), we found that the predicted stability of simulated protein sequences at early evolutionary stages, when they carried only a few mutations relative to *T*_*0*_, overlapped with that of the real sequence datasets (approximately ranging from −25 to 100 kcal/mol for all the studied proteins) (Fig. 1). However, as the evolutionary distance from *T*_*0*_ increased, it predicted a systematic decline in protein stability, reflected by progressively higher Δ*G* for the simulated sequences. Of note, we generated mutants using comparative HM, and RMSD comparisons between modeled mutant structures and the initial *T*_*0*_ template revealed that the evolutionary trajectories retained structural similarity to *T*_*0*_ (RMSD < 1.5 along almost all trajectories (Fig. 2)). For the real sequences, we likewise observed minimal structural divergence relative to *T*_*0*_. This observation may reflect the conservation of secondary structure required to maintain protein function. However, the same pattern was also observed for randomly mutated sequences, which would be expected to accumulate substantially greater structural alterations.

**Fig. 3:**
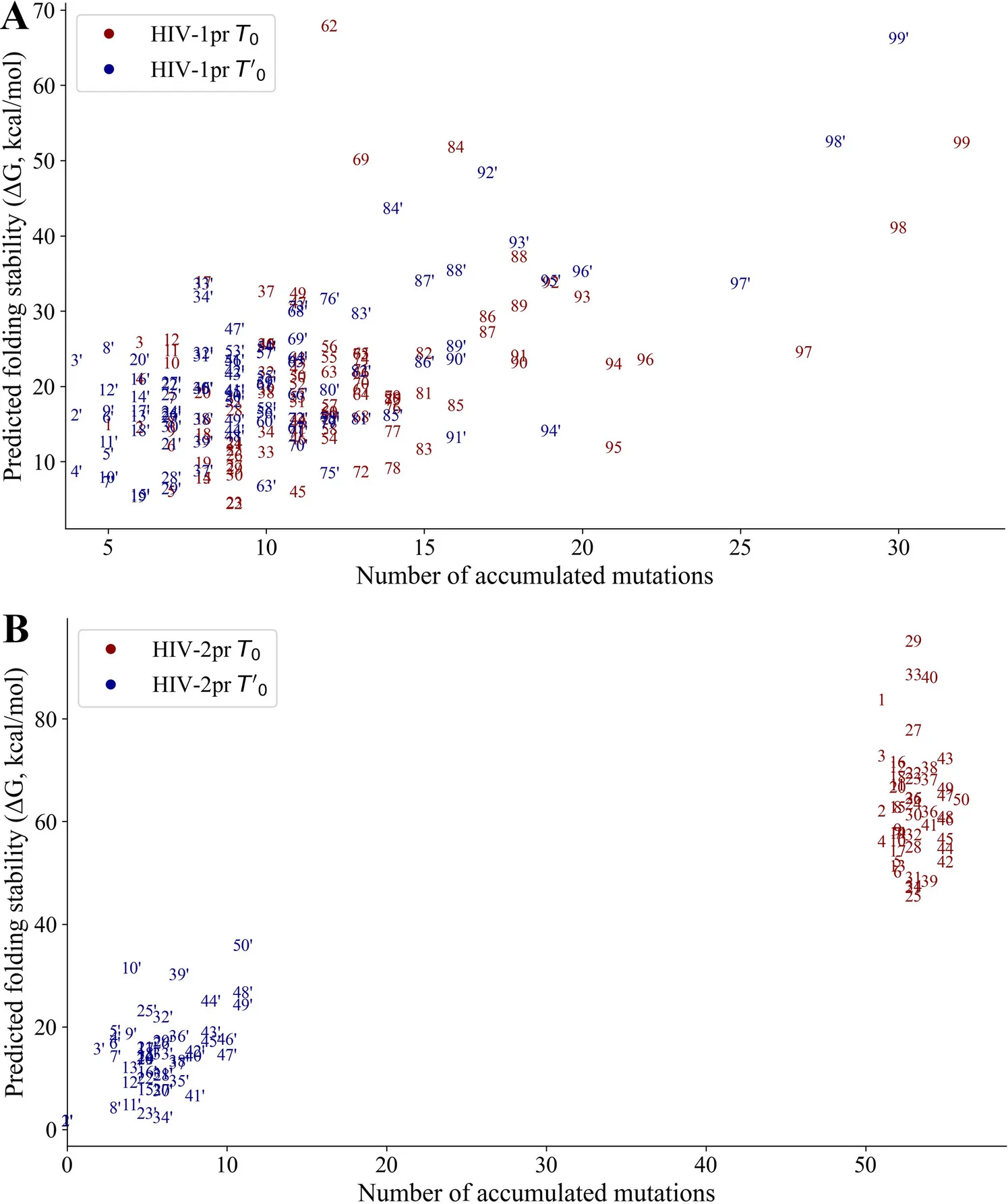
Influence of template choice on folding-stability estimates for real sequence data. Protein folding stability (Δ*G* predicted by *FoldX*) of real sequences using different templates (the original template *T*_*0*_ (red) and an alternative template *T′*_*0*_ (blue)) as a function of the number of accumulated substitutions relative to each template. Individual real sequences are identified by numerical labels, and primes (′) indicate predictions obtained using *T′*_*0*_. In panel **A**, the real sequences and the alternative template correspond to HIV-1pr, whereas in panel **B**, they correspond to HIV-2pr. In both panels, *T*_*0*_ serves as the common reference template, illustrating the influence of template choice on the predicted stability landscape

In addition, we evaluated the influence of the template structure on structure-based HM folding stability predictions. To this end, we repeated the *FoldX* calculations and compared the resulting predicted stabilities (Δ*GT*_*0*_* versus* Δ*GT′*_*0*_). The results showed that the same sequence could yield different predicted stability depending on the template used for homology modeling (Fig. 3), reflecting the important sensitivity of structure-based HM predictions to template selection. Using an alternative HIV-1 PDB template (Fig. 3A), we observed variations in the predicted stability in both directions, indicating that template selection is critical even when analyzing mutants with only minor sequence differences. In contrast, when analyzing HIV2pr (Fig. 3B), which is highly divergent from *T*_*0*_ (HIV-1 and HIV-2 proteases share only ~ 50% sequence identity), we found that the predicted stability of HIV2pr was higher than that of simulated sequences along the evolutionary trajectories carrying an equivalent number of mutations relative to *T*_*0*_. As expected, when the HIV2pr structure was used as the template (*T′*_*0*_), the predicted stability was substantially lower. These results further illustrate the strong influence of template selection and suggest that the use of inappropriate templates can reduce the accuracy of folding stability predictions (Fig. 3).

**Fig. 4:**
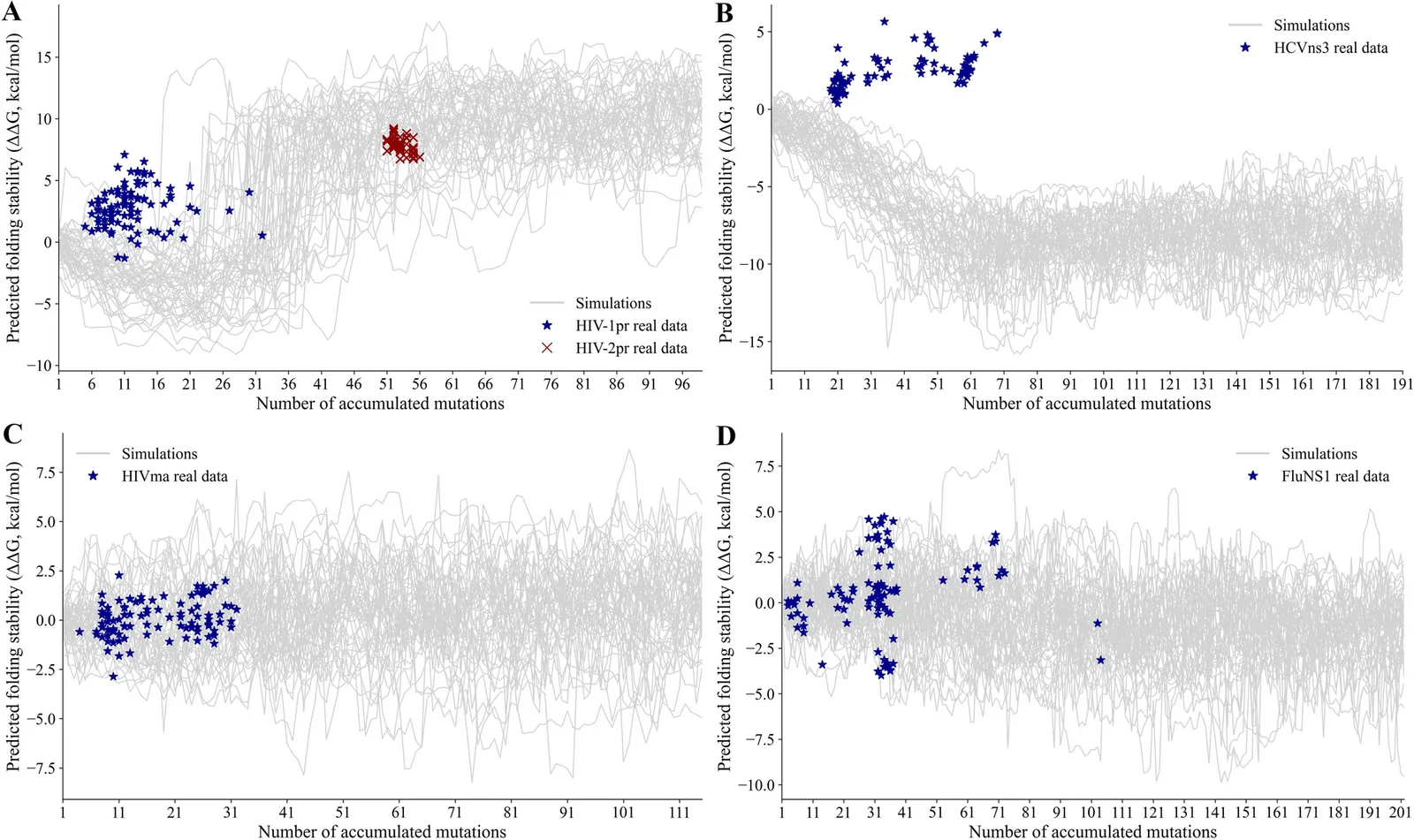
Protein folding stability of simulated mutational trajectories and real sequence data predicted using a sequence-based ML approach. Relationship between the number of accumulated mutations and protein folding stability (Δ*G*) predicted with *THPLM*. Panels show predictions for **A** HIVpr, **B** HCVns3, **C** HIVma, and **D** FluNS1 proteins. Simulated evolutionary trajectories are shown in gray, whereas real sequence data are represented by blue stars. In panel **A**, blue stars correspond to HIV-1pr sequences and red crosses to HIV-2pr sequences

**Fig. 5:**
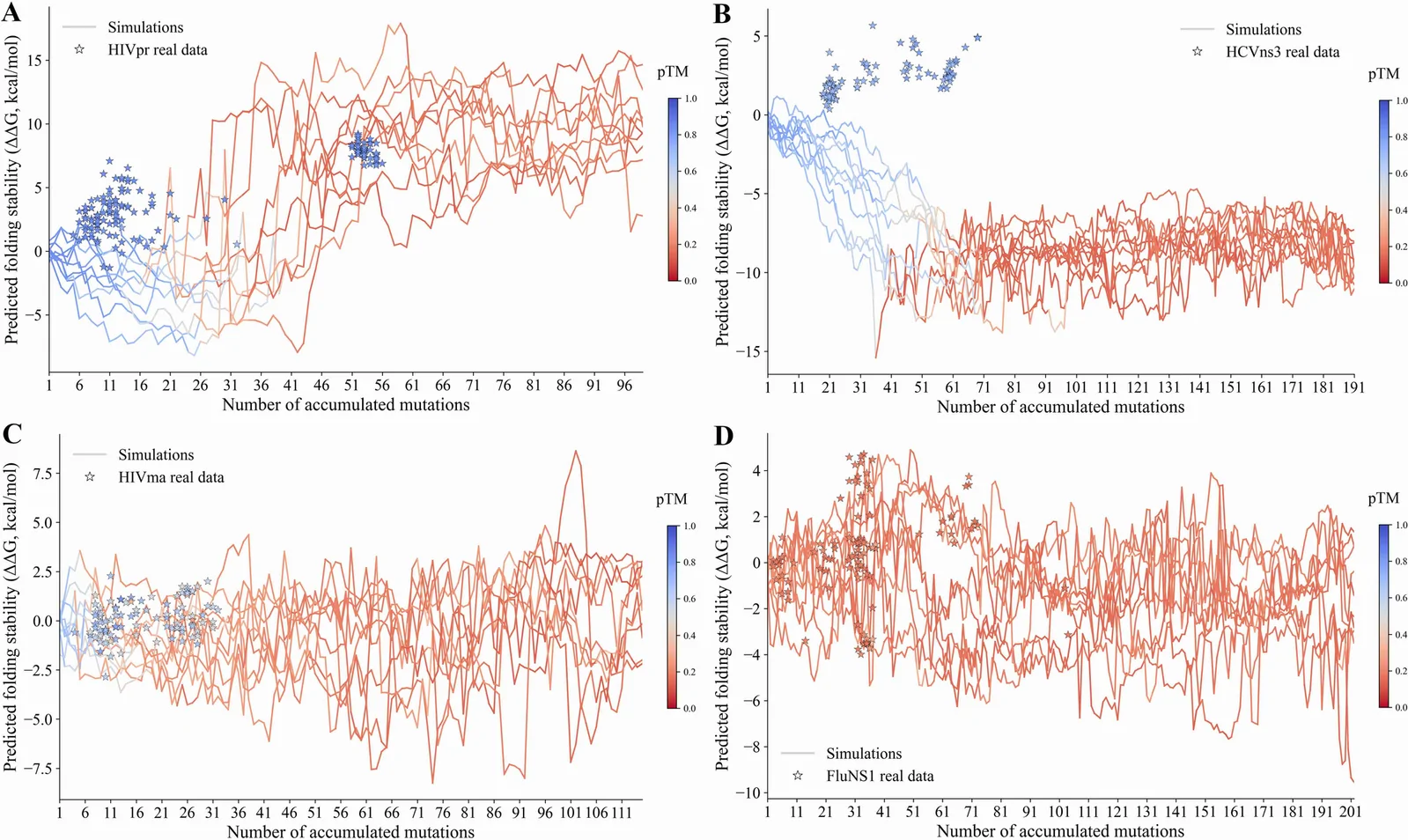
Prediction confidence of protein folding stability in simulated mutational trajectories and real sequence data using a sequence-based ML approach. Relationship between the number of accumulated mutations, protein folding stability (Δ*G* predicted by *THPLM*), and the predicted template modeling (pTM) score obtained with *ESMFold* for each simulated and real sequence. Panels show predictions for **A** HIVpr, **B** HCVns3, **C** HIVma, and **D** FluNS1 proteins. Simulated evolutionary trajectories are colored according to their pTM score (ranging from 0 to 1), with blue indicating higher prediction confidence. Real sequence data (stars) are shown using the same pTM color scale

Regarding the predictions obtained with the sequence-based ML framework (*THPLM*), the predicted stability of the real sequences fell within an interval of approximately −5 to 7.5 kcal/mol (Fig. 4), which was narrower than that obtained with *FoldX*. A key methodological difference between ML and HM approaches is that, whereas the latter rely on a structural template to generate mutant protein structures, the former represent proteins through learned sequence embeddings and do not require an explicitly specified structural template. Consequently, *THPLM* may provide more accurate folding-stability predictions for proteins with greater amino acid sequence divergence relative to *T*_*0*_. Accordingly, during the early stages of the HIVpr simulations, predicted stability was maintained or even showed a slight increase in some trajectories (Fig. 4A), while pTM scores remained high (Fig. 5A). However, after accumulating a relatively large number of mutations (20–30), the predicted stability decreased and then remained approximately constant throughout the remainder of the trajectory (Fig. 4A), accompanied by a decline in pTM scores (Fig. 5A). The stability estimates obtained for real sequences closely related to *T*_*0*_ were slightly lower than those predicted for the corresponding simulated sequences (Fig. 4A). In contrast, the stability estimates for the more divergent HIV2pr sequences were similar to those obtained for simulated sequences carrying a comparable number of mutations relative to *T*_*0*_, while retaining high pTM scores (Fig. 5A). A similar pattern was observed for HCVns3, where the predicted stability decreased during the early stages of the evolutionary trajectories and subsequently remained approximately constant until the end of the trajectories (Fig. 4B), while confidence (pTM scores) declined after the accumulation of a relatively large number of mutations (40–50) (Fig. 5B). In this case, however, real sequences showed lower predicted folding stability than the corresponding simulated sequences throughout the evolutionary trajectories (Fig. 4B). For HIVma and FluNS1, predicted stability remained largely constant throughout the simulated trajectories, and the estimates obtained from the real sequences fell within the range predicted for the simulated data (Fig. 4C and D). Notably, confidence in the predictions was low for both proteins across all simulated trajectories, as reflected by the low pTM scores (Fig. 5C and D), and also for the real FluNS1 sequences (Fig. 5D). These results illustrate that folding-stability predictions can be problematic for certain proteins, including well-studied viral proteins. Overall, the performance of the method appears to be constrained more by the characteristics of the training data than by the level of sequence divergence itself, indicating protein-specific prediction behavior. We also generated structures for all variants obtained along the trajectories simulated with *THPML* using *ESMFold*. The RMSD between the structures predicted by *ESMFold* and the reference structure (*T*_*0*_) showed a marked increase (exceeding 15 Å) as mutations accumulated (Figure S1; Supplementary Material), suggesting substantial structural rearrangements.

## Discussion

### Marginal stability as a consequence of evolutionary adaptation and biophysical constraints

The stability predicted from the real sequences of all the studied proteins, regardless of the method used, showed fluctuations consistent with marginal stability. Although the accuracy of the stability prediction methods is discussed later, we first consider the factors that may underlie this phenomenon.

The observation of marginal stability across nearly all protein families, regardless of their size or complexity, has been explained by different mechanisms. From an evolutionary perspective, marginal stability arises from a dynamic equilibrium shaped by the balance among mutation, selection, and genetic drift [2, 5]. According to this view, most random mutations are destabilizing, pushing proteins toward unfolded states, upon which purifying selection acts as a filter that maintains the minimum level required for function. This process generates a stability buffer that allows proteins to tolerate functionally beneficial (or neutral) mutations that can be slightly destabilizing. In contrast, an alternative perspective proposes that marginal stability represents a universal biophysical constraint arising from the physical nature of folding specificity [37, 38]. In particular, these authors applied statistical mechanics and the principle of least action to the problem of protein folding, identifying a robust upper bound for the marginal stability of monomeric globular proteins, estimated at approximately 7.4 kcal/mol [37, 38]. This limit would arise from a quasi-equilibrium of forces at the global free-energy minimum and would be independent of evolutionary processes and post-translational chemical modifications. Notably, even large ribonucleoprotein complexes such as the ribosome showed the same stability constraints as small monomeric enzymes, suggesting that this phenomenon is scale-invariant [38]. Furthermore, that study showed that the distribution of ΔΔ*G* associated with single-point mutations is almost universally confined to a range of approximately ± 5 kcal/mol, which is consistent with the proposed upper bound [38]. According to this view, neutral evolution with respect to protein stability can therefore be regarded as a general phenomenon operating within a physically constrained stability range.

Although these theories have often been considered independently, they can be complementary (Table 1). A large-scale experimental analysis of 500 human protein domains revealed that the effects of mutations on protein stability are largely conserved across homologous domains [14]. More than 60% of pathogenic variants reduced protein stability, whereas burial depth predicted the magnitude of destabilization, supporting both the evolutionary conservation and biophysical constraint perspectives [14]. Emerging models have also begun to bridge these perspectives by linking evolutionary fitness signals to local physicochemical environments inferred from sequence information, indicating that evolutionary and biophysical constraints are complementary rather than competing explanations [9, 39]. Indeed, a recent analysis of DMS data showed that evolutionary information derived from multiple sequence alignments, particularly when combined with structural properties such as relative solvent accessibility, can achieve high predictive performance, supporting the view that marginal stability reflects, at least in part, evolutionary adaptation [40]. Similarly, ancestral sequence reconstruction combined with structural and dynamical modeling has revealed variation in protein stability associated with increased frequencies of small hydrophobic residues and hydrophobic contacts, thereby identifying specific biophysical constraints that shape evolutionary trajectories [41–44]. Nevertheless, understanding the causes of marginal stability remains a fundamental challenge because estimates of Δ*G* are highly method dependent. This issue is discussed in the following sections.

**Table 1:** Comparison of the main features of the mutation-selection-drift theory and the universal biophysical theory of protein marginal stability. The table summarizes the proposed determinants of protein folding stability, the expected stability ranges, and the underlying basis of the stability constraints

Feature	Mutation-selection-drift theory	Universal biophysical theory
Primary driver	Adaptive selection *versus* mutation pressure	Statistical thermodynamics of the protein fold
Stability range	Variable (5–15 kcal/mol)	Fixed upper bound (~ 7.4 kcal/mol)
Role of drift	Causes gradual degradation of stability	Operates within a physically defined margin
Fundaments of stability constraint	Population genetics and adaptation	Contact network entropy

### Rationale for using FoldX and THPLM frameworks for protein folding stability calculation

The *FoldX* [29, 45] and *THPLM* [30] frameworks implement widely used structure-based and sequence-based approaches for protein folding stability calculation, respectively, based on their complementary modeling, computational efficiency, superior performance compared with other methods, and broad applicability [46, 47]. *FoldX* estimates mutation-induced stability changes using an empirical energy function and a structural template, whereas *THPLM* leverages protein language model representations to predict stability changes directly from sequence. In this study, this combination enables us to examine how structural constraints and sequence-derived evolutionary information influence stability across increasingly divergent sequences. Alternative structure-based approaches, such as *Rosetta* [48], provide specific energy units that complicate comparisons with the free Gibbs energy. Molecular dynamics simulations (e.g., using *AMBER* [49]) offer more extensive conformational sampling and detailed physical representations of protein mutations, but require additional parameterization and modeling and involve substantial computational costs [50]. *THPLM* performed better than other sequence-based predictors, including *DDGun*, *INPS*, *SAEEFC*-*SEQ*, and *MUpro* [30]. Moreover, *THPLM* considers a more realistic parameterization compared to other methods (i.e., *PROSTATA* [51]). In this regard, *THPLM* explicitly computes the difference between wild-type and mutant sequence representations and incorporates both forward and reverse mutations during training, directly modeling the effect of the mutation rather than relying solely on downstream regression of individual embeddings. In summary, *FoldX* and *THPLM* provide appropriate and well-established representative frameworks for structure-based and sequence-based approaches, respectively, for computationally tractable protein folding stability calculation, allowing comparisons between these two currently available contrasting methodological paradigms. However, their selection was not intended to constitute a systematic benchmark of available stability prediction methods.

### Prediction of protein folding stability using structure-based HM approaches: the rigid-backbone trap and limited structural template availability

The effects of mutations on protein folding stability have traditionally been studied using homology modeling in combination with empirical force fields, as implemented in frameworks such as *FoldX* [28, 29, 45], which were parameterized using extensive collections of experimental ΔΔ*G* measurements. The energy functions implemented in these frameworks account for a variety of physicochemical interactions, including van der Waals interactions, hydrogen bonding, electrostatic interactions, and the solvation energies of both polar and apolar groups [29]. Because prediction accuracy also depends on the quality of the input structures, preprocessing protocols are commonly applied to resolve local steric clashes and optimize the orientation of residues whose electron densities are often ambiguous in X-ray crystallographic structures [29, 52]. These repair procedures identify residues with energetically unfavorable conformations and explore alternative rotamer combinations to identify local energy minima, thereby providing a more realistic starting point for Δ*G* calculations. Despite their speed and widespread use, these methods rely on the fundamental assumption that the polypeptide backbone remains fixed throughout the mutational process. Consequently, in CMS, as sequences diverge from the original template (*T*_*0*_), the mutated amino acid sequences are forced to adopt the geometry of the wild-type fold [15]. This generates a form of structural memory in which the RMSD of the mutant models relative to the wild-type structure remains artificially low (often < 1.5 Å, Fig. 2), even after substantial sequence divergence (Fig. 1). Thus, the observed structural conservation is a consequence of the modeling framework rather than a reflection of biological constraints. In nature, the accumulation of mutations is often accompanied by backbone rearrangements that accommodate new side chains and interactions. Such conformational divergence is consistent with the well-established observation that structural similarity decreases non-linearly with sequence divergence, with substantial structural divergence emerging below approximately 30% sequence identity [53]. Consistent with this limitation, *FoldX* has been reported to correctly identify approximately 69% of destabilizing mutations but only 29% of stabilizing mutations [47]. Consequently, structure-based explorations of broad fitness landscapes may be biased because backbone relaxation is not allowed. Consistent with this limitation, our analyses showed that the resulting underestimation of structural tolerance became increasingly pronounced as simulated sequences accumulated mutations and diverged from the reference template (Fig. 1). This limitation is particularly relevant for hypervariable loop regions and protein-protein interfaces, where conformational plasticity is often essential for function [54, 55]. Therefore, structure-based HM approaches for predicting protein folding stability should better account for the interplay among backbone flexibility, structural constraints, and epistatic interactions, whereby the effect of a mutation may depend on the presence of mutations at other positions [56, 57].

Another limitation of these approaches is the restricted availability of experimental structures that can serve as reliable templates. Although multiple structures of different variants of the same protein may be available in the PDB, they generally correspond to highly similar sequences and therefore fail to capture the extensive sequence diversity observed in natural populations [58]. In this regard, extensive benchmarking has shown that the reliability of ΔΔ*G* predictions depends on the sequence identity between the target protein and the structural template. In particular, a critical threshold of approximately 40% sequence identity has been proposed [59, 60]. Below this threshold, alignment errors become sufficiently large to compromise the accuracy of stability predictions. When sequence identity falls below 30%, the target and template proteins may adopt different folds, rendering structure-based stability calculations highly unreliable. Even above the 40% threshold, modeling noise can still affect stability calculations. In particular, we observed substantial differences in the predicted folding stability from HIV-2 sequences depending on whether the HIV-2 T*′*_*0*_ structure or the HIV-1 *T*_*0*_ structure was used as the modeling template, despite the two proteins sharing approximately 40–50% sequence identity (Fig. 3B). More unexpectedly, using two alternative HIV-1 templates (*T*_*0*_ and *T′*_*0*_), which share approximately 85% sequence identity, also affected the stability predictions. This suggests that the choice of template structure can significantly influence studies of the effects of mutations. Consistent with these observations, studies comparing Δ*G* predictions derived from experimentally determined PDB structures and corresponding homology models have reported prediction errors up to 1.5-fold higher for modeled structures, even when sequence identity exceeds 50% [61]. Accurate stability calculations require highly precise placement of both backbone atoms and buried side chains, the latter being particularly sensitive to the subtle structural perturbations introduced during the modeling process [59]. Systematic benchmarking has likewise shown that structure-based predictors can exhibit substantial performance deterioration when applied to homology models generated from templates below approximately 40% of sequence identity, with the effect being particularly pronounced for stabilizing and solvent-exposed mutations [60]. This provides an independent computational basis for interpreting the template-dependent differences observed in the simulations.

### Prediction of protein folding stability using sequence-based ML approaches: the training-space limitation and the prediction from artificial structures with sequence-based ML

To circumvent the bottlenecks associated with protein folding-stability predictions based on structural modeling, sequence-based ML approaches, such as *THPLM*, have introduced a paradigm shift in stability prediction [30]. The strength of these approaches lies in their capacity to capture evolutionary information and global sequence context without requiring an explicit structural template. Because PLMs are trained on millions of unannotated protein sequences using a masked-language-modeling objective, they internalize statistical patterns of protein architecture, including residue-residue contacts and functional motifs [62]. As a result, *THPLM* achieves high accuracy on single-point mutation benchmarks and often outperforms traditional structure-based methods [30]. However, these approaches have their own methodological limitations. Whereas structure-based HM methods are limited by structural rigidity, sequence-based ML methods are constrained by the boundaries of their training data, limiting their capacity to explore regions of the fitness landscape that are poorly represented in the training data [63]. For example, *THPLM* is trained primarily on experimental data from databases such as *ProThermDB*, which are heavily biased toward single-point mutations in well-folded proteins. Consequently, its ability to explore regions of the fitness landscape that are poorly represented in the training set may be limited. In this regard, we found that the predicted stability of real sequences fell within an interval of approximately −5 to 7.5 kcal/mol. Notably, this range overlaps with both the experimentally observed range of marginal stability (approximately 5–15 kcal/mol [17, 18]) and the theoretical upper limit of marginal stability [37, 38]. In contrast, the predicted stability of the simulated sequences ranged from −15 to 20 kcal/mol (Fig. 4). Consistently, we observed a decline in prediction confidence after the initial stages of the simulated evolutionary trajectories (Fig. 5). Of particular concern was FluNS1, for which neither the reference sequence *T*_*0*_ nor the naturally occurring variants reached acceptable confidence levels (Fig. 5D).

However, the low confidence of sequence-based ML methods is not only due to the training data. A further, and particularly important, aspect concerns the reliability of the embeddings themselves. Prabakaran and Bromberg (2026) [64] introduced the first generalized framework for quantifying uncertainty in protein-language-model embeddings. By comparing how a PLM encodes natural proteins *versus* randomly generated synthetic sequences in latent space, the authors defined a “random neighbor score,” corresponding to the proportion of non-biological synthetic sequences among the nearest neighbors of a protein embedding. Their analysis revealed that low-quality embeddings, characterized by high random neighbor scores, display vector properties indistinguishable from those of randomly generated sequences, indicating that the model has failed to encode biologically meaningful information. When applied to the highly mutated artificial trajectories explored in CMS, as simulated sequences diverge from the natural protein space on which ESM-2 was trained, their embeddings are increasingly likely to fall into low-quality regions of the latent space. Under these conditions, ΔΔ*G* predictions generated by tools such as *THPLM* may become less reliable and may reflect limitations of the training distribution rather than the genuine thermodynamic properties of the protein sequence. The absence of uncertainty estimates in the evaluation of the effects of mutations remains a recognized limitation of current approaches. Systematic evaluations have shown that transformer-based architectures typically predict the effects of multiple mutations through additive combinations without providing measures of prediction uncertainty, preventing users from assessing prediction confidence when models are applied outside their training distribution [56, 65]. Additional biases have also been identified. PLMs exhibit systematic biases arising from unequal sequence sampling, which can lead to species-dependent likelihood scores unrelated to intrinsic protein properties and may therefore confound thermostability predictions [66]. Indeed, pTM confidence values for NS1 real data were lower than other proteins real data, suggesting a poor representation of this protein in the training dataset. Furthermore, even when fine-tuned on very large datasets, these models showed limited generalization to larger protein scaffolds, indicating that the diversity of the training data, rather than its size, is a key determinant of predictive performance [63, 67]. This interpretation is consistent with systematic analyses showing that the performance of protein-stability predictors can vary substantially across datasets and mutation types, with selection biases in the experimental data used for training or parameterization contributing substantially to apparent differences in predictive performance [68]. More generally, the intrinsic noise and distribution of experimentally measured ΔΔ*G* values also impose limits on the maximum achievable predictive correlation, making comparisons across datasets potentially misleading [69]. Finally, we generated structures for the *THPLM*-simulated trajectories using *ESMFold* and calculated their RMSD relative to ${T}_{0}$. We found that as the number of mutations increased, the RMSD increased accordingly, reaching values up to 17–25 Å. Because *THPLM* predictions are not constrained by an initial structural template, this indicates that structural rearrangements become increasingly pronounced as mutations accumulate. This substantial deviation from the original template suggests that highly mutated variants might adopt entirely different folds, calling into question both the validity of the predicted stability and whether the simulated protein retains its functional identity. Even though *ESMFold* is a different program than *THPLM*, it should be noted that both are based on the same embedding architecture (Meta ESM-2 [33]). Therefore, decreases in pTM scores and predicted stability may partly reflect a common signal associated with sequence divergence from the training space, as also suggested by the increase in RMSD. Because *THPLM* is trained using experimental thermodynamic data from *ProThermDB* [70], which provide information not directly captured by *ESMFold*, the pTM score can be used as a rough indicator of sequence structural plausibility. However, it should be interpreted cautiously, as it is not completely independent of the stability predictions.

### Conclusions and future perspectives

Altogether, the observation that natural proteins exhibit marginal stability is a biological fact, yet methodological limitations currently hamper our ability to define precise thresholds of functional stability. Our comparative analyses suggest that, in practice, marginal stability should not be regarded as a fixed universal limit, such as 7.4 kcal/mol. Rather, its range may vary among protein families and according to the assumptions underlying the prediction methodologies employed. We showed that current computational approaches for predicting protein folding stability have important limitations when applied to sequences that accumulate mutations beyond the diversity represented in the underlying reference datasets, whether experimental structures in HM-based approaches or sequence embeddings in ML-based approaches. As a result, stability predictions for highly divergent sequences should be interpreted with caution. Future progress will likely depend on reducing biases associated with limited training data and incorporating additional sources of biological information, including structural flexibility, multi-site epistasis, and even non-functional regions of sequence space. More broadly, studies of protein fitness should integrate predicted stability with other relevant molecular properties, such as intermolecular interactions, solubility, conformational entropy, transition-state kinetics, and the structural flexibility required for biological activity. Such integrative approaches may provide a more realistic understanding of how proteins evolve under the constraints of marginal stability.

## Supplementary Information

Below is the link to the electronic supplementary material.

Supplementary Material (PDF 246 KB)

## Data Availability

The mutational trajectory data and the protein folding stability predictions are available from the Zenodo repository at the URL https://doi.org/10.5281/zenodo.20733067.
