# Supplementary material for: On the limits of marginal protein folding stability: methodological goals and drawbacks
Source: J Mol Model. 2026 Oct 27;32(11):448. doi: 10.1007/s00894-026-07034-6 (PMC13714046; doi:10.1007/s00894-026-07034-6)
Supplement: Supplementary file 1 [file 894_2026_7034_MOESM1_ESM.pdf]

Supplementary Material

On the limits of marginal protein folding stability: Methodological goals and drawbacks

The supplementary material includes Table S1, Figure S1 and the cited references.

Supplementary Table

Table S1. Template protein structures used in this study. The table shows the selected PDB structures used either as reference templates (T_0) for the simulated evolutionary trajectories or as alternative templates (T'_0) to evaluate the effect of template choice on homology modelling and protein stability predictions.

Organism and protein family	PDB code	Template
HIV protease (HIVpr)	1KJH [1]	T_0
Hepatitis C virus ns3 (HCVns3)	3M5N [2]	T_0
HIV matrix (HIVma)	1HIW [3]	T_0
Influenza virus NS1(FluNS1)	4OPH [4]	T_0
HIV-1 protease (HIV-1pr)	3IXO [5]	T'_0
HIV-2 protease (HIV-2pr)	3S45 [6]	T'_0

Supplementary Figure

Figure S1. Structural divergence of simulated mutational trajectories and real sequence data relative to T_0 using a structure-based ML approach for protein folding stability prediction. Relationship among the number of accumulated mutations, protein folding stability (ΔG predicted by *THPLM*), and the RMSD of structures predicted by *ESMFold* relative to the reference structure (T_0) for simulated and real sequence data. Panels show predictions for (A) HIVpr, (B) HCVns3, (C) HIVma, and (D) FluNS1 proteins. Simulated evolutionary trajectories are colored according to their RMSD from the reference structure (T_0), with red indicating higher structural divergence. Real sequence data (stars) are shown using the same RMSD color scale relative to T_0.

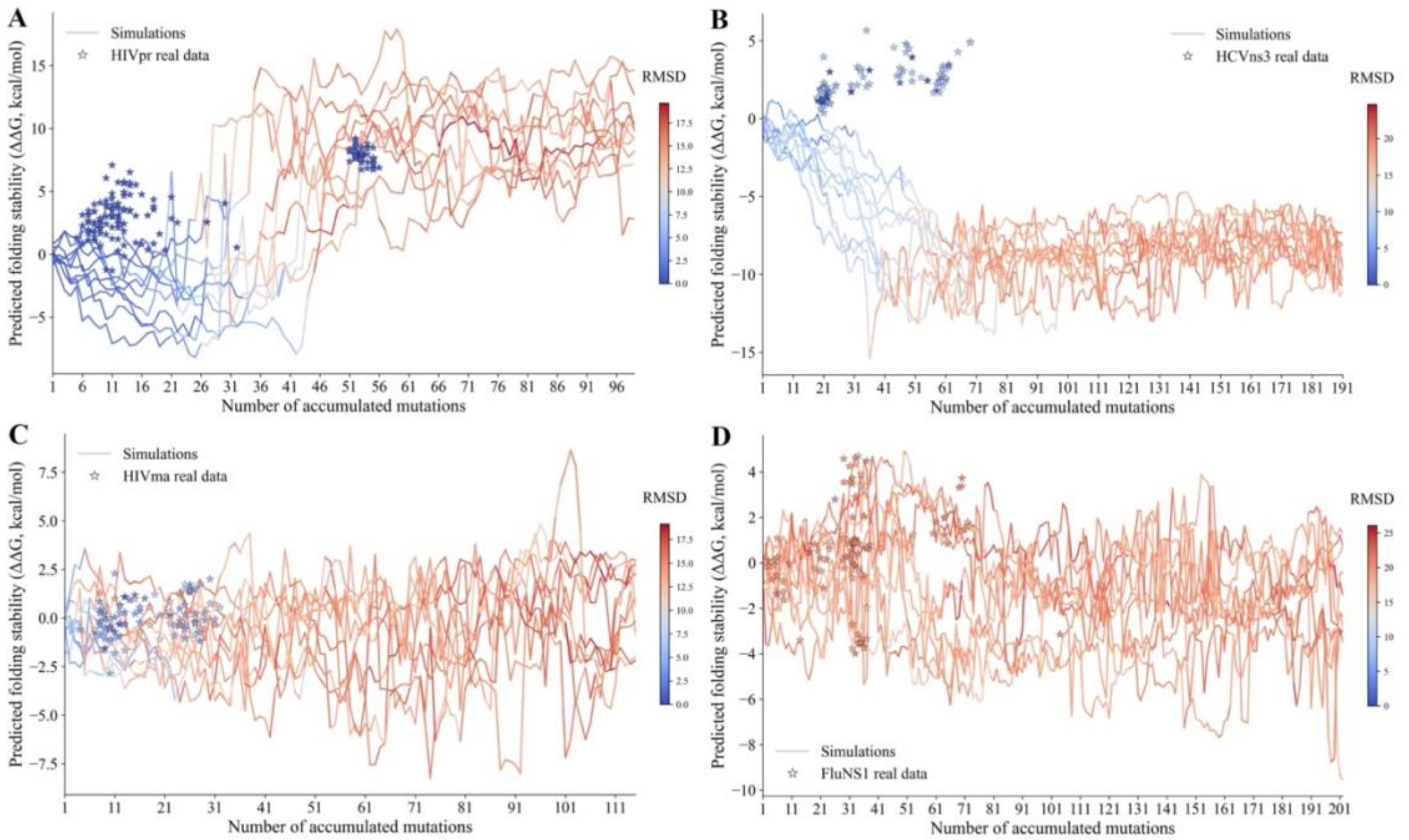

References cited in the Supplementary Material

1. Prabu-Jeyabalan M, Nalivaika E, Schiffer CA (2002) Substrate Shape Determines Specificity of Recognition for HIV-1 Protease: Analysis of Crystal Structures of Six Substrate Complexes. 10:369–381. https://doi.org/https://doi.org/10.1016/S0969-2126(02)00720-7

2. Romano KP, Ali A, Royer WE, Schiffer CA (2010) Drug resistance against HCV NS3/4A inhibitors is defined by the balance of substrate recognition versus inhibitor binding. Proc Natl Acad Sci 107:20986–20991. https://doi.org/10.1073/pnas.1006370107

3. Hill CP, Worthylake D, Bancroft DP, et al (1996) Crystal structures of the trimeric human immunodeficiency virus type 1 matrix protein: implications for membrane association and assembly. Proc Natl Acad Sci 93:3099–3104. https://doi.org/10.1073/pnas.93.7.3099

4. Carrillo B, Choi J-M, Bornholdt ZA, et al (2014) The Influenza A Virus Protein NS1 Displays Structural Polymorphism. J Virol 88:4113–4122. https://doi.org/10.1128/JVI.03692-13

5. Robbins AH, Coman RM, Bracho-Sanchez E, et al (2010) Structure of the unbound form of HIV-1 subtype A protease: comparison with unbound forms of proteases from other HIV subtypes. Acta Crystallogr D Biol Crystallogr 66:233–242. https://doi.org/10.1107/S0907444909054298

6. Tie Y, Wang Y, Boross PI, et al (2012) Critical differences in HIV-1 and HIV-2 protease specificity for clinical inhibitors. Protein Sci 21:339–350. https://doi.org/10.1002/pro.2019
